# Integrins α_v_β_3_ and α_v_β_5_ as prognostic, diagnostic, and therapeutic targets in gastric cancer

**DOI:** 10.1007/s10120-014-0435-2

**Published:** 2014-10-15

**Authors:** Christine Böger, Viktoria S. Warneke, Hans-Michael Behrens, Holger Kalthoff, Simon L. Goodman, Thomas Becker, Christoph Röcken

**Affiliations:** 1 Department of Pathology, Christian Albrechts University, Arnold-Heller-Str. 3, Haus 14, 24105 Kiel, Germany; 2 Department of Experimental Cancer Research, Christian Albrechts University, Kiel, Germany; 3 Oncology Platform, Department of Translational and Biomarkers Research, Merck KGaA, Darmstadt, Germany; 4 Department of General Surgery and Thoracic Surgery, Christian Albrechts University, Kiel, Germany

**Keywords:** Integrins, α_v_β_3_, α_v_β_5_, Immunohistochemistry, Gastric cancer

## Abstract

**Background:**

We investigated the expression of two α_v_ integrins, α_v_β_3_ and α_v_β_5_, in gastric cancer (GC) by testing the following hypotheses: that these molecules are expressed in GC; that they are implicated in GC biology; that they help to distinguish between the two major histological subtypes of GC, according to Laurén; and that they are prognostically relevant.

**Methods:**

Formalin-fixed and paraffin-embedded tissue samples from 482 GC samples were stained immunohistochemically using rabbit monoclonal antibodies directed against α_v_β_3_ (EM22703) and α_v_β_5_ (EM09902). Immunostaining of tumor, stroma, and endothelial cells was evaluated separately by the quantity and intensity, generating an immunoreactivity score. The immunoreactivity score of both antibodies was correlated with clinicopathology data and patient survival.

**Results:**

Each integrin was expressed in at least one tumor component in all GCs. Both were expressed significantly more often in the intestinal phenotype according to Laurén. Moreover, patients who grouped as “positive” for expression of α_v_β_3_ on endothelial cells, and patients with an intestinal type GC, grouped as “negative” for expression of α_v_β_5_ on stroma cells, had significantly longer survival. The expression of α_v_β_5_ on stroma cells was confirmed to be an independent prognostic factor of intestinal-type GC.

**Conclusion:**

The expression of α_v_β_3_ and α_v_β_5_ in at least one tumor component in all GC samples is an interesting new result that should form a basis for further investigations; for example, regarding selective integrin antagonists and the value of α_v_β_3_ and α_v_β_5_ as putative prognostic biomarkers. Moreover, both markers might be helpful in the routine classification of GC subtypes.

**Electronic supplementary material:**

The online version of this article (doi:10.1007/s10120-014-0435-2) contains supplementary material, which is available to authorized users.

## Introduction

In recent decades we have witnessed major advances in the understanding of the epidemiology, pathology, and pathogenesis of gastric cancer (GC). Infection with *Helicobacter pylori* or Epstein–Barr virus and dietary and lifestyle factors contribute to the risk of developing GC. This progress has been accompanied by the introduction of chemotherapy for GC, which is evolving continuously and which has improved patients’ survival [1–3]. Evidence is accumulating that patient prognosis and treatment response depend not only on the tumor stage but also on tumor-specific alterations of both gene expression and various signaling pathways. The two major histological subtypes of GC according to Laurén, diffuse-type and intestinal-type GC, have distinct tumor dissemination patterns and show diverse pathogeneses and expression profiles, likely resulting from molecular differences in tumor epithelial and stroma cells [4, 5]. Although the distinction between diffuse and intestinal subtype in GC has prognostic significance, it is still widely neglected in patient-tailored treatment of GC [6, 7].

Integrins are a family of 24 heterodimeric, multifunctional glycoproteins. As cell adhesion molecules and cell surface receptors, they mediate cell-to-cell and cell to extracellular matrix interactions, and are involved in a great variety of physiological and pathological processes [8]. They are composed of an α subunit, and a β subunit that connect to the cytoskeleton and interact with multiple signaling pathways; the α–β combination determines integrin ligand binding specificity and intracellular signaling [9]. Integrins are important regulators of differentiation, tumor growth, survival, migration, and invasion. In malignant tumors, they are involved in several processes that characterize the tumor phenotype [10]. Several integrin heterodimers have already been shown to be involved in GC biology and to have a significant value as prognostic markers. An increased expression of integrin α_v_β_6_ is linked significantly with reduced survival, lymph node metastasis, and the number of cancer-associated fibroblasts, and integrin α_5_β_1_ is described to be significantly associated with tumor differentiation, TNM stage, and recurrence [11–15]. Recently, integrins, particularly α_v_β_3_ and α_v_β_5_, have been recognized as putative targets for the treatment of several cancers, which has spurred research on integrins in cancer biology [16–19]. Thus, the characterization of integrin distribution in human tumors is of great interest. At present little is known about the expression of integrins α_v_β_3_ and α_v_β_5_ in GC, mainly owing to the lack of antibodies suitable for use on formalin-fixed and paraffin-embedded (FFPE) tissue [20]. Only two studies to date have focused on integrins α_v_β_3_ and α_v_β_5_ in GC. Those studies differ significantly from our study, as they investigated only 19 and 55 cases, respectively, and relied on frozen tissue sections. Also, owing to the small number of cases, they were unable to correlate the expression pattern of α_v_β_3_ and α_v_β_5_ in GC with clinicopathological patient characteristics [12, 21].

Recently, comprehensive molecular characterization including whole-genome sequencing was performed in GC and nontumor pairs for integrative genomic analysis of GC [22, 23]: 20 of 26 genes of the integrin subunits were deregulated in GC pathways, involving also cell adherens junctions, angiogenesis, and focal adhesion. Thus, deregulation of integrin expression may be a tumor-biological hallmark of GC or its specific subtypes. However, data on integrin expression on a protein level in GC are still sparse, and validation of genomic data is urgently needed. Here we investigated the expression of α_v_β_3_ and α_v_β_5_ in GC on the protein level, examining the following questions:

1. Are integrins expressed in GC?
2. Are integrins implicated in GC biology?
3. Do integrins discriminate the GC subtypes?
4. Is the expression of integrins prognostically relevant?

## Materials and methods

### Study population

From the archive of the Institute of Pathology, University Hospital Kiel, we identified 611 Caucasian patients who underwent either total or partial gastrectomy for adenocarcinoma of the stomach or esophagogastric junction between 1997 and 2009. The following patient characteristics were retrieved: type of surgery, age at diagnosis, gender, tumor size, tumor localization, tumor type, tumor grade, depth of invasion, number of lymph nodes resected, and number of lymph nodes with metastases. Each resected specimen underwent gross sectioning and histological examination by surgical pathologists. The date of patient death was obtained from the *Epidemiological Cancer Registry* of the state of Schleswig–Holstein, Germany. Follow-up data for those patients who were still alive were retrieved from hospital records and general practitioners. Ethical approval was obtained from the local ethical review board (D 453/10). All patient data were pseudonymized prior to inclusion in the study. Tissue was included if (1) an adenocarcinoma of the stomach or esophagogastric junction was confirmed histologically, (2) the date of death or survival data were available, and (3) the overall tumor mass was large enough to get three tissue microarray (TMA) punches. Exclusion criteria were defined as follows: (1) histological examination identified a tumor type other than adenocarcinoma; (2) patients had undergone perioperative chemotherapy or radiotherapy; and (3) the date of the patient’s death or survival data had not been recorded.

In total, 482 patients fulfilled all study inclusion criteria. The clinicopathological patient characteristics are summarized in Table 1. In accordance with Laurén, an intestinal type was found in 247 patients (51.2 %), a diffuse type was found in 152 patients (31.5 %), a mixed type was found in 30 patients (6.2 %), and an unclassifiable type was found in 53 patients (11.0 %).

**Table 1:** Clinico-pathological patient characteristics

Patients (*n*)	482
Age (years)	
Mean (range) ± SD	67.9 (33–92) ± 11.1
Median	68.0
Gender, *n* (%)	
Men	297 (61.6)
Women	185 (38.4)
Lauren phenotype, *n* (%)	
Intestinal	247 (51.2)
Diffuse	152 (31.5)
Mixed	30 (6.2)
Unclassified	53 (11.0)
Localization, *n* (%)	
Proximal	149 (30.9)
Distal	333 (69.1)
pT-category, *n* (%)	
pT1a	13 (2.7)
pT1b	49 (10.2)
pT2	56 (11.6)
pT3	190 (39.4)
pT4a	134 (27.8)
pT4b	40 (8.3)
pN-category, *n* (valid %)	
pN0	138 (28.8)
pN1	67 (14.0)
pN2	85 (17.7)
pN3 (a/b)	189 (39.5)
Missing	3
UICC stage (7th ed.), *n* (valid %)	
IA	49 (10.4)
IB	32 (6.8)
IIA	58 (12.3)
IIB	47 (9.9)
IIIA	55 (11.6)
IIIB	83 (17.5)
IIIC	66 (14.0)
IV	83 (17.5)
Missing	9
Stage (“Kiel proposal”), *n* (valid %)	
I	49 (10.2)
II	84 (17.5)
IIIA	49 (10.2)
IIIB	153 (31.9)
IV	145 (30.2)
Missing	2
Resection margin, *n* (%)	
pR0	403 (83.6)
pR1/2	54 (11.2)
pRx	25 (5.2)
Lymphatic invasion, *n* (valid %)	
pL0	228 (48.8)
pL1	239 (51.2)
Missing	15
Venous invasion, *n* (valid %)	
pV0	413 (88.8)
pV1	52 (11.2)
Missing	17
Resected lymph nodes, *n* (valid %)	
≤16	195 (41.6)
>16	274 (58.4)
Missing	13
Positive lymph nodes, *n* (valid %)	
0	134 (28.6)
1–2	67 (14.3)
≥3	267 (57.1)
Missing	14
Tumor grade, *n* (valid %)	
G1/G2	111 (23.7)
G3/G4	357 (76.3)
Missing	14
Follow-up data, *n* (%)	
Dead	335 (69.5)
Alive	131 (27.2)
Unknown	16 (3.3)

### Histology

Tissue specimens were fixed in formalin and embedded in paraffin. Deparaffinized sections were stained with hematoxylin and eosin. Histological reexamination of primary tissue sections was done for all cases to ensure if inclusion criteria were confirmed. Tumors were classified according to the Laurén classification [4] and were reexamined by two surgical pathologists. The pTNM stage of all study patients was determined according to the seventh edition of the Union for International Cancer Control (UICC) guidelines [24] and the recent proposal (Kiel stage) of Warneke et al. [25].

### Tissue microarray construction

FFPE tissue samples were used to generate TMAs as described previously [26]. Briefly, three morphologically representative regions of the paraffin “donor” blocks (tumor) were chosen, and tissue cylinders of 1.5-mm diameter were punched from these areas. Afterwards, the tissue cylinders were inserted into a new “recipient” paraffin block using a custom-built instrument (Beecher Instruments, Silver Spring, MD, USA). The new recipient paraffin blocks were warmed in a 60 °C heating cabinet for 7 min to create a sufficient bond between the tumor tissue and the recipient block paraffin. Then, 2.5-μm-thick serial sections were obtained from the new recipient paraffin blocks, dried in a 60 °C heating cabinet for 6 h, and stored in polystyrene slide storage boxes at 8 °C until use.

### Immunohistochemistry

For immunohistochemistry we used two monoclonal rabbit antibodies, directed against α_v_β_3_ (EM22703) and α_v_β_5_ (EM09902). The biochemical specificity of the antibodies against integrins, which were used in this study, was precisely defined previously [20]. All immunoreactions for validation used the Ventana BenchMark XT automated slide staining system using the reaction buffer ULTRA LCS, EZ Prep (75 °C; 4 min), protease 2 (12 min), UV inhibitor, ultraView Universal DAB, hematoxylin II, and bluing reagent (all reagents from Roche Diagnostics, Mannheim, Germany). Antibodies were diluted in antibody diluent (Zytomed Systems, Berlin, Germany) and were applied at 10 µg/ml for 36 min at 36 °C (anti-α_v_β_3_), or 0.1 µg/ml for 40 min at 40 °C (anti-α_v_β_5_). To determine the optimal antibody dilution, kidney sections were stained with serial dilutions of the primary antibodies (1 ng to 100 μg/ml). Distinctive staining patterns of α_v_β_3_ (mainly glomerular) and α_v_β_5_ (glomerular and descending tubuli) were used as reference positive controls for calibration and initial titration of the antibodies. Rabbit IgG preimmune sera (Abcam, Cambridge, UK) served as negative controls. Negative and positive controls were applied in parallel for each staining series. Additionally, we conducted immunohistochemistry with a monoclonal antibody directed against E-cadherin as previously described [6]. The E-cadherin staining results were correlated with those of α_v_β_3_ and α_v_β_5_.

### Study design

TMA sections from each tumor were stained with antibodies directed against α_v_β_3_ and α_v_β_5_. The staining results were correlated with clinicopathology and survival data.

### Evaluation of immunostaining

The quantity, intensity, and localization of immunoreactivity of both antibodies were evaluated by applying an immunoreactivity scoring system. Immunoreactivity was evaluated separately for tumor cells, stroma cells, and endothelial cells. Stroma cells included all cells of the tumor stroma (e.g. fibroblasts), and excluded endothelial cells, which were evaluated separately.

The previously described immunoreactivity scoring system [27] consisted of two components. Category A rated the percentage of immunoreactive cells and was graded as 0 (negative), 1 (up to 10 % positive cells), 2 (10–50 % positive cells), 3 (51–80 % positive cells), and 4 (81–100 % positive cells). Category B documented the intensity of immunostaining as 0 (no immunostaining), 1 (weak), 2 (moderate), or 3 (strong). The addition of category A and category B resulted in an immunoreactivity score (IRS), with was separately applied for tumor cells and stroma cells. The IRS ranged from 0 to 7 for tumor cells and from 0 to 7 for stroma cells. The intensity of the endothelial immunoreaction was rated as 0 (negative), 1 (weak), 2 (moderate), or 3 (strong).

### Statistical analysis

Statistical analyses were done using SPSS 20.0 (IBM, New York, NY, USA). For comparison purposes, the IRS for tumor cells, the IRS for stroma cells, and the endothelial immunoreaction were partitioned at the median, and patients below the median were classified as “negative.” Median overall survival was determined using the Kaplan–Meier method, and the log-rank test was used to determine significance. To investigate the prognostic relevance, we included all variables having *p* < 0.100 in multivariate analysis using a Cox regression model and the backward logistic regression method (*p*_in_ and *p*_out_ = 0.05) to reduce the model to the independent variables. The significance of correlation between clinicopathological parameters and each antigen’s IRS was tested using Fisher’s exact test. For parameters of ordinal scale (T category, N category, tumor stage), we applied Kendall’s tau test instead. To account for the effects of multiple testing, we applied the explorative Simes (Benjamini–Hochberg) procedure [28]. We considered *p* ≤ 0.05 statistically significant. No adjustments were made.

## Results

### Staining results

**Fig. 1:**
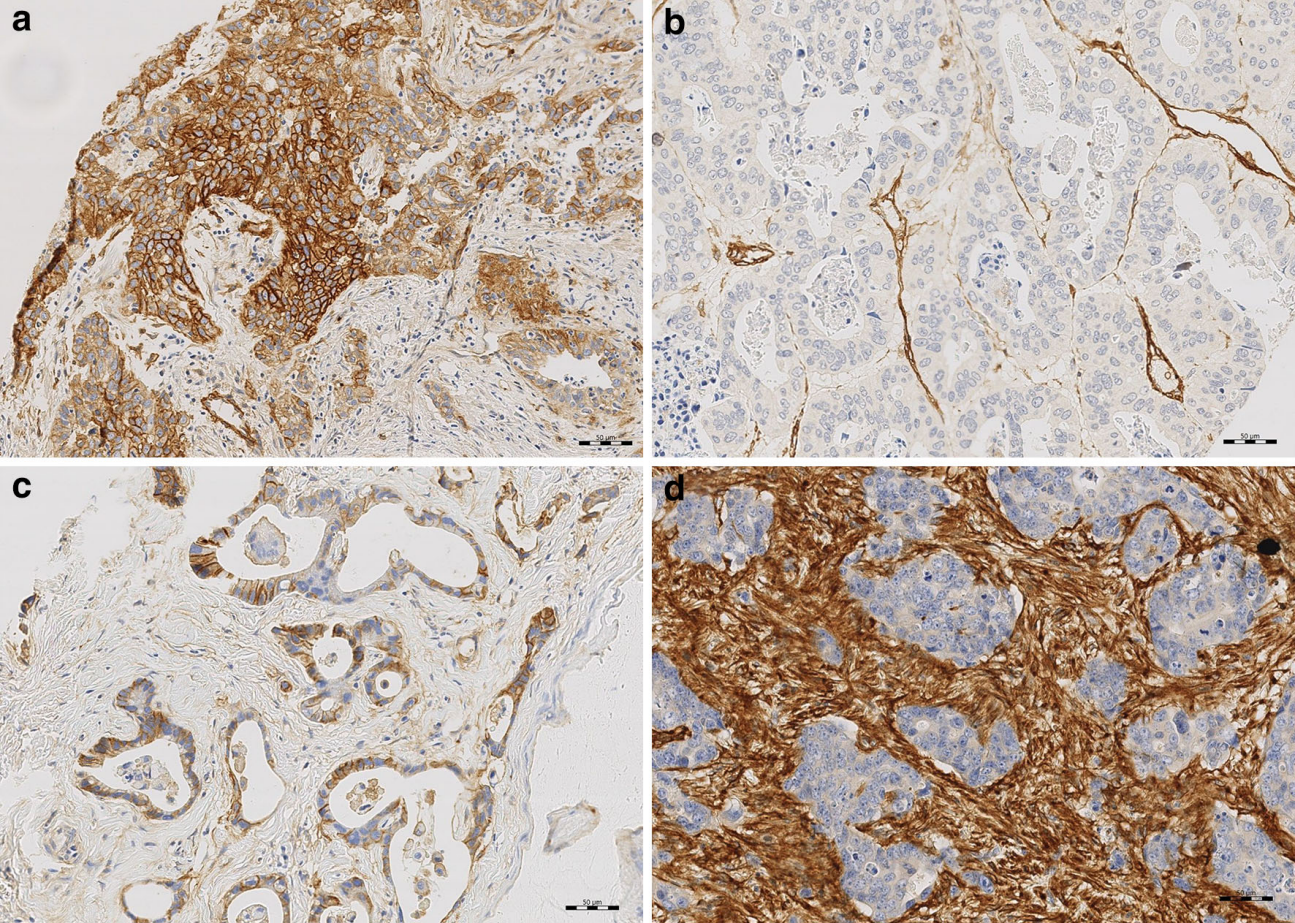
Expression of α_v_β_3_ and α_v_β_5_ in gastric carcinoma. This figure illustrates gastric carcinomas of the intestinal type according to Laurén with a strong membranous expression of α_v_β_3_ on tumor cells (**a**) and on endothelial cells (**b**), a moderate membranous expression of α_v_β_5_ on tumor cells (**c**), and a strong α_v_β_5_ expression on stroma cells (**d**). Original magnification ×200

Both α_v_β_3_ and α_v_β_5_ were expressed in at least one tumor component in all GC samples investigated (Fig. 1). Expression in tumor cells was mainly membranous. In cases with a strong membranous and/or stromal expression, an additional light cytoplasmic staining was observed.

### Integrin α_v_β_3_

Integrin α_v_β_3_ was expressed in 119 of 457 cases (26.0 %) in tumor cells. The percentage of stained tumor cells ranged from grade 0 to grade 4 (median grade 0), the staining intensity ranged from 0 (no immunostaining) to 3 (strong immunoreaction; median 0), and the tumor cell IRS ranged from 0 to 7 (median 0). Dichotomized by the median, 119 cases (26.0 %) were classified as positive and 338 cases (74.0 %) were classified as negative. In stroma cells, α_v_β_3_ was expressed in 420 of 457 cases (91.9 %). The percentage of stained stroma cells ranged from grade 0 to grade 4 (median grade 1), the staining intensity ranged from 0 to 3 (median 1), and the stroma cell IRS ranged from 0 to 7 (median 3). Dichotomized by the median, 247 cases (54.0 %) were classified as positive and 210 cases (46.0 %) were classified as negative. Integrin α_v_β_3_ was expressed in endothelial cells in all cases (456 of 456; 100 %). Staining intensity ranged from 1 to 3 (median 2). Dichotomized by the median, 363 cases (79.6 %) were classified as positive and 93 cases (20.4 %) were classified as negative.

### Integrin α_v_β_5_

Integrin α_v_β_5_ was expressed in 299 of 453 cases (66.0 %) in tumor cells. The percentage of stained tumor cells ranged from grade 0 to grade 4 (median grade 2), the staining intensity ranged from 0 to 3 (median 1), and the tumor cell IRS ranged from 0 to 6 (median 3). Dichotomized by the median, 246 cases (54.3 %) were classified as positive and 207 cases (45.7 %) were classified as negative. In stroma cells, α_v_β_5_ was expressed in all cases (454 of 454; 100 %). The percentage of stained stroma cells ranged from grade 1 to grade 4 (median grade 2), the staining intensity ranged from 1 to 4 (median 2), and the stroma cell IRS ranged from 2 to 7 (median 4). Dichotomized by the median, 189 cases (41.6 %) were classified as positive and 265 cases (58.4 %) were classified as negative. The expression of α_v_β_5_ in endothelial cells could not be analyzed in 125 of 482 cases owing to a strong stromal immunoreaction. In the remaining 357 cases, α_v_β_5_ was expressed in endothelial cells in 274 cases (76.8 %). Staining intensity ranged from 0 to 2 (median 1). Dichotomized by the median, 274 cases (76.8 %) were classified as positive and 83 cases (23.2 %) were classified as negative.

### Clinicopathological correlation

Next we studied the correlation between the expression of the integrins and the clinicopathological patient characteristics. For this purpose, we split the IRS of each marker at the median into negative (at or below the median IRS) and positive (above the median IRS) cases. Significant correlations were found for gender, tumor type, tumor localization, T category, tumor stage according to the UICC and Kiel classifications, and tumor grade. Most interestingly, diffuse-type GC exhibited a significantly reduced expression of both integrins in tumor cells and in stroma cells compared with intestinal-type GC. There was no significant correlation between N category, venous invasion, or lymph vessel invasion and the expression of either tested marker. Co

mplete data are given in Table 2.

**Table 2:** Correlation between α_v_β_3_ and α_v_β_5_ expression in tumor cells, stroma cells and endothelium and clinico-pathological patient characteristics

All patients			α_v_β_3_						α_v_β_5_					
			Tumor cells		Stroma cells		Endothelium		Tumor cells		Stroma cells		Endothelium	
			Negative [n (%)]	Positive [n (%)]	Negative [n (%)]	Positive [n (%)]	Negative [n (%)]	Positive [n (%)]	Negative [n (%)]	Positive [n (%)]	Negative [n (%)]	Positive [n (%)]	Negative [n (%)]	Positive [n (%)]
Gender	*n*	*p*	457	0.514	457	0.387	456	0.634	453	0.175	454	**<0.001**	357	0.701
Men			204 (72.9)	76 (27.1)	124 (44.3)	156 (55.7)	55 (19.6)	225 (80.4)	120 (43.0)	159 (57.0)	142 (50.7)	138 (49.3)	52 (24.2)	163 (75.8)
Women			134 (75.7)	43 (24.3)	86 (48.6)	91 (51.4)	38 (21.6)	138 (78.4)	87 (50.0)	87 (50.0)	123 (70.7)	51 (29.3)	31 (21.8)	111 (78.2)
Lauren phenotype	*n*	*p*	457	**0.005**	457	**0.004**	456	**0.003**	453	**<0.001**	454	**<0.001**	357	0.662
Intestinal			155 (66.5)	78 (33.5)	88 (37.8)	145 (62.2)	33 (14.2)	199 (85.8)	83 (35.5)	151 (64.5)	99 (42.3)	135 (57.7)	42 (24.6)	129 (75.4)
Diffuse			118 (81.9)	26 (18.1)	80 (55.6)	64 (44.4)	42 (29.2)	102 (70.8)	85 (60.7)	55 (39.3)	118 (83.7)	23 (16.3)	29 (22.1)	102 (77.9)
Mixed			24 (85.7)	4 (14.3)	13 (46.4)	15 (53.6)	8 (28.6)	20 (71.4)	14 (50.0)	14 (50.0)	20 (71.4)	8 (28.6)	3 (13.6)	19 (86.4)
Unclassified			41 (78.8)	11 (21.2)	29 (55.8)	23 (44.2)	10 (19.2)	42 (80.8)	25 (49.0)	26 (51.0)	28 (54.9)	23 (45.1)	9 (27.3)	24 (72.7)
Localization	*n*	*p*	457	0.732	457	0.213	456	0.319	453	**0.011**	454	**<0.001**	357	0.273
Proximal stomach			105 (72.9)	39 (27.1)	60 (41.7)	84 (58.3)	25 (17.5)	118 (82.5)	52 (36.6)	90 (63.4)	66 (46.5)	76 (53.5)	20 (19.2)	84 (80.8)
Distal stomach			233 (74.4)	80 (25.6)	150 (47.9)	163 (52.1)	68 (21.7)	245 (78.3)	155 (49.8)	156 (50.2)	199 (63.8)	113 (36.2)	63 (24.9)	190 (75.1)
pT-category	*n*	*p*	457	0.949	457	0.052	456	**0.005**	453	0.362	454	**<0.001**	357	0.495
pT1a			9 (75.0)	3 (25.0)	4 (33.3)	8 (66.7)	1 (8.3)	11 (91.7)	4 (50.0)	4 (50.0)	9 (100.0)	0 (0.0)	3 (37.5)	5 (62.5)
pT1b			28 (70.0)	12 (30.0)	10 (25.0)	30 (75.0)	2 (5.0)	38 (95.0)	15 (35.7)	27 (64.3)	27 (64.3)	15 (35.7)	11 (32.4)	23 (67.6)
pT2			42 (76.4)	13 (23.6)	24 (43.6)	31 (56.4)	10 (18.2)	45 (81.8)	23 (44.2)	29 (55.8)	26 (50.0)	26 (50.0)	7 (18.4)	31 (81.6)
pT3			133 (72.3)	51 (27.7)	85 (46.2)	99 (53.8)	34 (18.6)	149 (81.4)	81 (44.0)	103 (56.0)	101 (54.9)	83 (45.1)	35 (25.0)	105 (75.0)
pT4a			97 (76.4)	30 (23.6)	66 (52.0)	61 (48.0)	31 (24.4)	96 (75.6)	61 (47.3)	68 (52.7)	71 (55.0)	58 (45.0)	21 (20.4)	82 (79.6)
pT4b			26 (74.4)	10 (25.6)	21 (53.8)	18 (46.2)	15 (38.5)	24 (61.5)	23 (60.5)	15 (39.5)	31 (81.6)	7 (18.4)	6 (17.6)	28 (82.4)
pN-category	*n*	*p*	455	0.919	455	0.439	454	0.110	450	0.409	451	0.710	354	0.500
pN0			96 (75.0)	32 (25.0)	52 (40.6)	76 (59.4)	17 (13.4)	110 (86.6)	53 (42.4)	72 (57.6)	76 (60.8)	49 (39.2)	22 (22.2)	77 (77.8)
pN1			51 (77.3)	15 (22.7)	34 (51.5)	32 (48.5)	14 (21.2)	52 (78.8)	33 (50.8)	32 (49.2)	41 (62.1)	25 (37.9)	14 (28.0)	36 (72.0)
pN2			57 (74.0)	20 (26.0)	35 (45.5)	42 (54.5)	19 (24.7)	58 (75.3)	40 (52.6)	36 (47.4)	42 (55.3)	34 (44.7)	10 (16.9)	49 (83.1)
pN3 (a/b)			134 (72.8)	50 (27.2)	89 (48.4)	95 (51.6)	43 (23.4)	141 (76.6)	81 (44.0)	103 (56.0)	103 (56.0)	81 (44.0)	37 (25.3)	109 (74.7)
Stage (UICC**)**	*n*	*p*	449	0.773	449	**0.011**	448	**0.037**	444	0.899	445	0.751	350	0.182
IA			27 (65.9)	14 (34.1)	10 (24.4)	31 (75.6)	3 (7.3)	38 (92.7)	18 (43.9)	23 (56.1)	29 (70.7)	12 (29.3)	12 (34.3)	23 (65.7)
IB			26 (83.9)	5 (16.1)	10 (32.3)	21 (67.7)	2 (6.5)	29 (93.5)	11 (39.3)	17 (60.7)	17 (58.6)	12 (41.4)	4 (18.2)	18 (81.8)
IIA			43 (78.2)	12 (21.8)	27 (49.1)	28 (50.9)	9 (16.7)	45 (83.3)	23 (42.6)	31 (57.4)	29 (53.7)	25 (46.3)	5 (12.8)	34 (87.2)
IIB			33 (70.2)	14 (29.8)	25 (53.2)	22 (46.8)	9 (19.1)	38 (80.9)	24 (52.2)	22 (47.8)	26 (56.5)	20 (43.5)	10 (29.4)	24 (70.6)
IIIA			39 (76.5)	12 (23.5)	28 (54.9)	23 (45.1)	12 (23.5)	39 (76.5)	25 (49.0)	26 (51.9)	28 (54.9)	23 (45.1)	9 (23.1)	30 (76.9)
IIIB			59 (72.8)	22 (27.2)	32 (39.5)	49 (60.5)	18 (22.2)	63 (77.8)	36 (43.9)	46 (56.1)	45 (54.9)	37 (45.1)	18 (27.3)	48 (72.7)
IIIC			48 (72.7)	18 (27.3)	38 (57.6)	28 (42.4)	17 (25.8)	49 (74.2)	34 (51.5)	32 (48.5)	41 (62.1)	25 (37.9)	16 (29.6)	38 (70.4)
IV			57 (74.0)	20 (26.0)	35 (45.5)	42 (54.5)	23 (29.9)	54 (70.1)	33 (43.4)	43 (56.6)	43 (56.6)	33 (43.4)	9 (14.8)	52 (85.2)
“Kiel”-stage	*n*	*p*	456	0.689	456	**0.050**	455	**0.013**	451	0.879	452	0.512	355	0.210
I			27 (65.9)	14 (34.1)	10 (24.4)	31 (75.6)	3 (7.3)	38 (92.7)	18 (43.9)	23 (56.1)	29 (70.7)	12 (29.3)	12 (34.3)	23 (65.7)
II			64 (78.0)	18 (22.0)	38 (46.3)	44 (53.7)	10 (12.3)	71 (87.7)	33 (41.2)	47 (58.8)	44 (55.0)	36 (45.0)	9 (15.0)	51 (85.0)
IIIA			37 (75.5)	12 (24.5)	25 (51.0)	24 (49.0)	9 (18.4)	40 (81.6)	24 (50.0)	24 (50.0)	29 (59.2)	20 (40.8)	9 (25.0)	27 (75.0)
IIIB			106 (73.1)	39 (26.9)	67 (46.2)	78 (53.8)	33 (22.8)	112 (77.2)	67 (46.5)	77 (53.5)	81 (56.2)	63 (43.8)	30 (26.8)	82 (73.2)
IV			104 (74.8)	35 (25.2)	70 (50.4)	69 (49.6)	38 (27.3)	101 (72.7)	65 (47.1)	73 (52.9)	80 (58.0)	58 (42.0)	23 (20.5)	89 (79.5)
Lymphatic invasion	*n*	*p*	444	0.453	444	0.392	443	0.347	439	0.701	440	0.847	346	0.703
pL0			160 (75.1)	53 (24.9)	100 (46.9)	113 (53.1)	39 (18.4)	173 (81.6)	98 (46.4)	113 (53.6)	124 (58.5)	88 (41.5)	37 (22.2)	130 (77.8)
pL1			166 (71.9)	65 (28.1)	99 (42.9)	132 (57.1)	51 (22.1)	180 (77.9)	101 (44.3)	127 (55.7)	131 (57.5)	97 (42.5)	43 (24.0)	136 (76.0)
Venous invasion	*n*	*p*	442	0.733	442	0.227	441	1.000	437	0.051	438	0.648	345	1.000
pV0			290 (73.8)	103 (26.2)	181 (46.1)	212 (53.9)	80 (20.4)	312 (79.6)	181 (46.8)	206 (53.2)	224 (57.7)	164 (42.3)	71 (23.0)	238 (77.0)
pV1			35 (71.4)	14 (28.6)	18 (36.7)	31 (63.3)	10 (20.4)	39 (79.6)	16 (32.0)	34 (68.0)	31 (62.0)	19 (38.0)	8 (22.2)	28 (77.8)
Tumor grade	*n*	*p*	445	**0.041**	445	0.091	445	**<0.001**	441	0.114	442	**0.002**	347	0.536
G1/G2			67 (65.7)	35 (34.3)	39 (38.2)	63 (61.8)	7 (6.9)	95 (93.1)	40 (38.8)	63 (61.2)	46 (44.7)	57 (55.3)	19 (26.4)	53 (73.6)
G3/G4			261 (76.1)	82 (23.9)	164 (47.8)	179 (52.2)	80 (23.3)	263 (76.7)	163 (48.2)	175 (51.8)	211 (62.2)	128 (37.8)	63 (22.9)	212 (77.1)
Survival	*n*	*p*	441	0.727	441	0.177	440	**0.024**	437	0.846	438	0.125	344	0.95
Median ± SD			14.6 ± 1.1	17.5 ± 3.9	16.9 ± 2.0	13.9 ± 1.3	12.1 ± 2.5	15.9 ± 1.2	16.5 ± 1.9	13.6 ± 1.4	17.1 ± 2.0	13.4 ± 1.6	17.1 ± 3.1	14.7 ± 1.2
[95 %CI]			[12.4–16.6]	[9.7–25.3]	[12.8–20.8]	[11.5–16.5]	[7.2–17.0]	[13.5–18.3]	[12.9–20.2]	[10.9–16.3]	[13.0–21.1]	[10.3–16.5]	[11.1–23.0]	[12.3–17.1]

*p* values printet in bold denote statistically significant correlations

*n* numbers of patients. *p* *p* value, *SD* standard deviation

Subgroup analyses of intestinal-type and diffuse-type GC showed that the endothelial expression of α_v_β_3_ in intestinal-type GC correlated significantly with the tumor grading: α_v_β_3_ was more often expressed in G1/G2 tumors than in G3/G4 tumors. Moreover, the stromal expression of α_v_β_5_ in intestinal-type GC correlated significantly with gender, the T category, and the tumor stage according to the Kiel classification. By contrast, there was no significant correlation between the expression of either marker and the clinicopathological patient characteristics in diffuse-type GC. Complete data are shown in Online Resource 1 and Online Resource 2.

The expression of E-cadherin in tumor cells correlated significantly with the expression of α_v_β_3_ in tumor cells (*p* < 0.001) and stroma cells (*p* = 0.006) as well as with the expression of α_v_β_5_ in tumor cells (*p* = 0.017). There was no significant correlation between the expression of E-cadherin and the expression of α_v_β_3_ in endothelial cells (*p* = 0.141) or the expression of α_v_β_5_ in stroma cells (*p* = 1.000) or endothelial cells (*p* = 0.885). Complete data on E-cadherin evaluation and the staining results are given in Online Resource 3.

### Prognostic significance

**Fig. 2:**
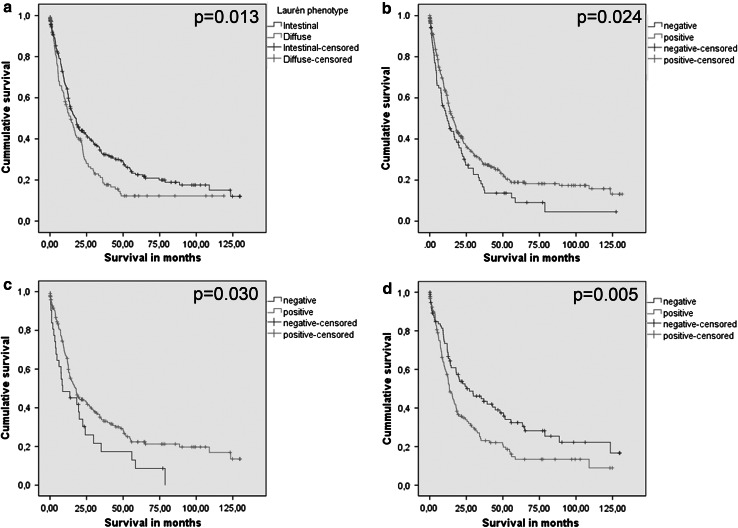
Kaplan–Meier curves for intestinal-type versus diffuse-type gastric carcinoma according to Laurén (**a**), α_v_β_3_ expression on endothelial cells in the entire cohort (**b**), α_v_β_3_ expression on endothelial cells in intestinal-type gastric carcinoma (**c**), and α_v_β_5_ expression on stroma cells in intestinal-type gastric carcinoma (**d**)

Patient prognosis of the entire cohort significantly depended on patient age, Laurén phenotype, T category, N category, lymphatic invasion, venous invasion, tumor grade, and UICC stage and Kiel stage (data not shown). Patients who were grouped as “positive” for expression of α_v_β_3_ on endothelial cells had significantly longer survival compared with patients with a “negative” α_v_β_3_ expression in endothelial cells (Table 2, Fig. 2).

The subgroup analyses of intestinal-type and diffuse-type GC showed that this also applied to patients with an intestinal-type GC: these patients had significantly longer survival if they were “positive” for expression of α_v_β_3_ on endothelial cells, compared with patients with a “negative” α_v_β_3_ expression. Patients with an intestinal-type GC grouped as “positive” for expression of α_v_β_5_ on stroma cells had significantly shorter survival than patients grouped as “negative” (Online Resource 1).

There were no other significant correlations between survival data and the expression of both markers.

### Explorative multivariate analysis

Explorative multivariate survival analysis was done with all parameters which had *p* < 0.100 in univariate survival analysis. For the entire study population and the diffuse-type subgroup, T category and N category were found to be highly significantly independent prognosticators of patient survival. The subgroup analysis of intestinal-type GC confirmed the independent prognostic significance of T category, N category, lymphatic invasion, and α_v_β_5_ expression on stroma cells (Table 3).

**Table 3:** Explorative multivariate survival analysis

Subgroup	Input parameters	Independent parameters kept in model	*p* value	HR (95 % CI)
All cases	Laurén phenotype			
	T-category	T-category	<0.001	1.031 (1.016–1.046)
	N-category	N-category	<0.001	1.041 (1.030–1.052)
	Lymphatic invasion			
	Venous invasion			
	Tumor grade			
	α_v_β_3_ expression on endothelial cells			
Intestinal type	T-category	T-category	0.026	1.024 (1.003–1.045)
	N-category	N-category	<0.001	1.029 (1.014–1.044)
	Lymphatic invasion	Lymphatic invasion	0.026	1.554 (1.054–2.289)
	Tumor grade			
	α_v_β_3_ expression on endothelial cells			
	α_v_β_5_ expression on stroma cells	α_v_β_5_ expression on stroma cells	0.043	1.417 (1.010–1.989)
Diffuse type	T-category	T-category	<0.001	1.067 (1.035–1.100)
	N-category	N-category	0.001	1.032 (1.014–1.051)
	Lymphatic invasion			
	Venous invasion			

A Cox regression was performed with all parameters as input which had *p* < 0.100 in univariate survival analysis.Tumor stage (Union for International Cancer Control stage and Kiel stage) was excluded from the model

*HR* hazard ratio

^a^The 95% confidence interval is given in *parentheses*.

## Discussion

GC is a heterogeneous disease, which still leads cancer deaths worldwide [29]. During recent years, evidence has accumulated indicating that patient prognosis and treatment response depend not only on tumor stage, but also on the expression and tumor-specific alteration of intracellular signaling pathways. Different treatment strategies are needed to specifically target the aberrant cancer signaling pathways in GC [30, 31].

Integrins α_v_β_3_ and α_v_β_5_ are at the focus of several oncologic investigations [32–39]. Integrins drive diverse intracellular signaling cascades, and so are involved in a great variety of physiological and pathological processes. They influence tumor cell proliferation, tumor cell movement, and cell survival in vivo and in vitro, and their involvement in multiple signaling pathways is crucial for tumor progression. This all suggests that integrins may be targets for the treatment of cancer, and this has spurred integrin research in cancer biology [9, 40, 41]. Some concepts for pharmacological treatment based on the inhibition of integrins already exist [18, 19], but implementation of a therapeutic strategy demands a robust verification of integrin expression in different tumors.

Here, for the first time we have investigated the expression of α_v_β_3_ and α_v_β_5_ in a large cohort of GC patients. Our primary observations are that:

1. Integrins α_v_β_3_ and α_v_β_5_ were expressed in at least one tumor component of all GC samples, which in general suggests that GC might be an interesting target for further studies on integrin-antagonistic cancer therapy.
2. A positive α_v_β_3_ status and a positive α_v_β_5_ status was observed significantly more often in intestinal-type GC than in diffuse-type GC. Intestinal-type GC is known to have a better outcome than diffuse-type GC [6]. We observed that a positive α_v_β_3_ status showed statistically significant correlations with several clinicopathological patient characteristics that are known to be associated with a better outcome, such as a minor pT category, a minor UICC/Kiel stage, and a better tumor grading (G1/G2). Indeed, a positive α_v_β_5_ status accompanies at least some of these characteristics. If we look more closely at these *p* values (*p* ≤ 0.05), it becomes clear that the distribution of the different subgroups is not as divergent as the statistics indicate. The subgroup analyses of intestinal-type and diffuse-type GC showed that only the endothelial expression of α_v_β_3_ correlated significantly with the tumor grading, and that only the stromal expression of α_v_β_5_ correlated significantly with the gender, the T category, and the tumor stage according to the Kiel classification. This indicates that the observed correlation between a positive α_v_β_3_ status and a positive α_v_β_5_ status and clinicopathological parameters that are associated with a better outcome is mainly caused by the increased expression of α_v_β_3_ and α_v_β_5_ in intestinal-type GC compared with diffuse-type GC. On the basis of these observations, one may speculate that both markers may be suitable to aid histological classification of GC.
3. Patients with an increased expression of α_v_β_3_ in endothelial cells, and patients with an intestinal-type GC “negative” for α_v_β_5_ had significantly longer survival. Moreover, α_v_β_5_ expression on stroma cells of intestinal-type GC was confirmed to be an independent prognostic factor. This interesting result is notable, as it seems that at least α_v_β_5_ has potential value as a prognostic biomarker for GC. Nevertheless, the significance and the clinicopathological relevance of these findings remain unclear and need to be addressed in further investigations.

Another interesting finding was the predominant expression of α_v_β_3_ and α_v_β_5_ in stromal and endothelial cells. There is evidence that intratumoral stroma is a predictor of survival in patients with GC [42]. Moreover, the proven correlation between the expression of both markers with E-cadherin confirms the well-known involvement of integrins in cell adhesion signaling [43, 44].

In other tumor entities, a high expression level of other α_v_ integrins has been described to be associated with tumor progression and worse survival, which is partly contradictory to our results. Previous studies mainly compared the expression level in neoplastic versus nonneoplastic tissue. In endometrial cancer, cervical squamous cell carcinoma, and serous epithelial ovarian carcinoma, an upregulation of integrin α_v_β_6_ was described in tumor tissue compared with normal cycling endometrium or nonneoplastic epithelia [45–47]. In colorectal cancer, the overexpression of α_v_ correlated significantly with poor prognosis [48]. In our study, we did not compare expression levels in nonneoplastic versus neoplastic tissue, but focused on the differential expression of α_v_β_3_ and α_v_β_5_ in the diverse cellular components of the neoplastic tissue compartment. Moreover, we used two other antibodies, with potentially different reactivity profiles in GC, than those that were used previously.

However, our study shows that the tumor-biological significance of integrins is not restricted to their expression by tumor cells. It extends into the intratumoral stroma and tumor vessels, and furthermore, may also depend by as yet unknown mechanisms on the histological phenotype. The differential expression of integrins in the tumor stroma of different GC tumor types somewhat supports the general observation that the tumor stroma is highly variable; for example, with or without pronounced desmoplasia. This is a particular hallmark of diffuse-type GC. We hypothesize that the diffuse type, with its poorly cohesive growth pattern, might be the result of decreased integrin expression of both tumor and stroma cells. Further studies of this topic may be productive.

One methodical issue in our study was that the pretreatment procedure for both antibodies was relatively intense. Both antibodies have previously been shown to deliver concordant staining results in frozen sections and FFPE tissue. Regarding FFPE tissue, even small deviations of the pretreatment temperature led to a decreased staining intensity and quality during manual staining of anti-α_v_β_3_ and anti-α_v_β_5_ [49]. Such hazards can be minimized by using fully automated staining systems, as done in the present study, and as designed for these antibodies [20]. Nevertheless, possible incomplete antigen retrieval or partial destruction of epitopes during the rather aggressive pretreatment has to be generally considered.

In conclusion, this study is the first extensive longitudinal investigation of the expression of integrins α_v_β_3_ and α_v_β_5_ in GC. Our data support recent whole genome sequencing data and suggest that GC is an interesting indication for further investigations of selective integrin antagonists, and that both α_v_β_3_ and α_v_β_5_ are selectively expressed in different GC classes, and might be valuable in classification of GC subtypes. Furthermore, it is clear that in GC at least α_v_β_5_ has potential value as a prognostic biomarker, and that both α_v_β_3_ and α_v_β_5_ might even be considered as novel therapeutic targets. Further investigations are needed, which, also in consideration of the comparison of integrin expression in tumor and nontumor tissue, might lead to additional information regarding the potential value of integrins α_v_β_3_ and α_v_β_5_ as diagnostic and prognostic biomarkers.

## Electronic supplementary material

Below is the link to the electronic supplementary material.

Supplementary material 1 (PDF 52 kb)

Supplementary material 2 (PDF 51 kb)

Supplementary material 3 (PDF 51 kb)
