# Supplementary material for: Integrins αvβ3 and αvβ5 as prognostic, diagnostic, and therapeutic targets in gastric cancer
Source: Gastric Cancer. 2014 Oct 15;18(4):784–95. doi: 10.1007/s10120-014-0435-2 (PMC4572058; doi:10.1007/s10120-014-0435-2)
Supplement: Supplementary file 1 — Supplementary material 1 (PDF 52 kb) [file 10120_2014_435_MOESM1_ESM.pdf]

Supplemental Table 1   Correlation between integrin expression in intestinal type gastric cancer and clinico-pathological patient characteristics

Intestinal Phenotype			αvβ3						αvβ5					
			Tumor Cells		Stroma Cells		Endothelium		Tumor Cells		Stroma Cells		Endothelium	
			Negative [n (%)]	Positive [n (%)]	Negative [n (%)]	Positive [n (%)]	Negative [n (%)]	Positive [n (%)]	Negative [n (%)]	Positive [n (%)]	Negative [n (%)]	Positive [n (%)]	Negative [n (%)]	Positive [n (%)]
**Gender**	n	p	233	0.764	233	1.000	232	0.543	234	0.882	234	**0.029**	171	0.438
Men			108 (65.9)	56 (34.1)	62 (37.8)	102 (62.2)	25 (15.2)	139 (84.8)	58 (35.2)	107 (64.8)	62 (37.6)	103 (62.4)	32 (26.7)	88 (73.3)
Women			47 (68.1)	22 (31.9)	26 (37.7)	43 (62.3)	8 (11.8)	60 (88.2)	25 (36.2)	44 (63.8)	37 (53.6)	32 (46.4)	10 (19.6)	41 (80.4)
**Localization**	n	p	232	0.889	233	0.340	232	0.572	234	0.053	234	0.141	171	0.151
Proximal stomach			47 (48.9)	49 (51.0)	33 (34.0)	64 (66.0)	12 (12.5)	84 (87.5)	27 (28.1)	69 (71.9)	35 (36.5)	61 (63.5)	13 (18.6)	57 (81.4)
Distal stomach			65 (47.8)	71 (52.2)	55 (40.4)	81 (59.6)	21 (15.4)	115 (84.6)	56 (40.6)	82 (59.4)	64 (46.4)	74 (53.6)	29 (28.7)	72 (71.3)
**pT-category**	n	p	233	0.745	233	0.224	232	0.230	234	0.455	234	**0.043**	171	0.689
pT1a			2 (50.0)	2 (50.0)	0 (0.00)	4 (100)	0 (0.0)	4 (100)	2 (50.0)	2 (50.0)	4 (100.0)	0 (0.0)	1 (25.0)	3 (75.0)
pT1b			23 (67.6)	11 (32.4)	8 (23.5)	26 (76.5)	1 (2.9)	33 (97.1)	13 (37.1)	22 (62.9)	21 (60.0)	14 (40.0)	10 (35.7)	18 (64.3)
pT2			22 (64.7)	12 (35.3)	13 (38.2)	21 (71.8)	5 (14.7)	29 (85.3)	10 (31.3)	22 (68.8)	11 (34.4)	21 (65.6)	4 (19.0)	17 (81.0)
pT3			61 (62.9)	36 (37.1)	38 (39.2)	59 (60.8)	14 (14.6)	82 (85.4)	36 (37.1)	61 (62.9)	38 (39.2)	59 (60.8)	15 (22.7)	51 (77.3)
pT4a			40 (74.1)	14 (25.9)	25 (46.3)	29 (53.7)	11 (20.4)	43 (79.6)	16 (28.6)	40 (71.4)	21 (37.5)	35 (62.5)	11 (25.6)	32 (74.4)
pT4b			7 (70.0)	3 (30.0)	4 (40.0)	6 (60.0)	2 (20.0)	8 (80.0)	6 (60.0)	4 (40.0)	4 (40.0)	6 (60.0)	1 (11.1)	8 (88.9)
**pN-category**	n	p	230	0.654	230	0.548	230	0.846	230	0.400	230	0.074	167	0.826
pN0			53 (66.3)	27 (33.7)	27 (38.6)	53 (61.4)	8 (10.1)	71 (89.9)	30 (38.0)	49 (62.0)	41 (51.9)	38 (48.1)	15 (22.2)	41 (77.8)
pN1			24 (68.6)	11 (31.4)	15 (42.9)	20 (67.1)	5 (14.3)	30 (85.7)	13 (37.1)	22 (62.9)	14 (40.0)	21 (60.0)	8 (28.0)	15 (72.0)
pN2			28 (71.8)	11 (28.2)	17 (43.6)	22 (56.4)	7 (17.9)	32 (82.1)	18 (46.2)	21 (53.8)	15 (38.5)	24 (61.5)	4 (14.8)	23 (85.2)
pN3 (a/b)			36 (63.2)	19 (26.8)	33 (50.8)	32 (49.2)	13 (17.1)	63 (82.9)	22 (28.6)	55 (71.4)	25 (32.5)	52 (67.5)	15 (25.0)	45 (75.0)
**Stage (UICC)**	n	p	229	0.705	229	0.160	229	0.475	229	0.723	229	0.111	167	0.515
IA			19 (61.3)	12 (28.7)	7 (22.6)	24 (77.4)	1 (3.2)	30 (96.7)	14 (43.8)	18 (56.2)	21 (65.6)	11 (34.4)	10 (38.5)	16 (61.5)
IB			17 (77.3)	5 (22.7)	6 (27.3)	16 (72.7)	2 (4.5)	20 (90.9)	7 (35.0)	13 (65.0)	11 (55.0)	9 (45.0)	2 (15.4)	11 (84.6)
IIA			22 (66.7)	11 (32.3)	14 (41.2)	19 (58.8)	4 (12.5)	28 (87.5)	12 (36.4)	21 (63.6)	12 (36.4)	21 (63.6)	3 (14.3)	18 (85.7)
IIB			11 (50.0)	11 (50.0)	11 (50.0)	11 (50.0)	4 (18.2)	18 (81.8)	8 (36.4)	14 (63.6)	8 (36.4)	14 (63.6)	4 (33.3)	8 (66.7)
IIIA			19 (70.4)	8 (29.6)	15 (55.5)	12 (45.5)	4 (14.8)	23 (85.2)	12 (44.4)	15 (55.6)	11 (29.7)	16 (70.3)	5 (25.0)	15 (75.0)
IIIB			26 (66.7)	13 (32.3)	11 (39.5)	28 (60.5)	6 (15.4)	33 (84.6)	14 (46.7)	16 (53.3)	16 (40.0)	24 (60.0)	8 (24.2)	25 (75.8)
IIIC			17 (68.0)	8 (32.0)	11 (44.0)	14 (66.0)	5 (20.0)	20 (80.0)	7 (28.0)	18 (72.0)	8 (32.0)	17 (68.0)	6 (33.3)	12 (66.7)
IV			22 (73.3)	8 (26.7)	12 (40.0)	18 (60.0)	7 (23.3)	23 (76.7)	9 (30.0)	21 (70.0)	9 (30.0)	21 (70.0)	4 (16.7)	20 (83.3)
**"Kiel"-Stage**	n	p	232	0.849	232	0.449	231	0.176	232	0.751	232	**0.026**	169	0.274
I			19 (61.3)	12 (28.7)	7 (22.6)	24 (77.4)	1 (3.2)	30 (96.7)	14 (43.8)	18 (56.2)	21 (65.6)	11 (34.4)	10 (38.5)	16 (61.5)
II			33 (67.3)	16 (32.7)	20 (40.8)	29 (59.2)	6 (12.5)	42 (87.5)	16 (34.0)	31 (66.0)	21 (36.8)	26 (63.2)	4 (13.3)	26 (86.7)
IIIA			17 (62.9)	10 (37.1)	11 (40.7)	16 (59.3)	3 (11.1)	24 (88.9)	10 (37.0)	17 (63.0)	10 (37.0)	17 (63.0)	5 (31.3)	11 (68.7)
IIIB			47 (66.2)	24 (33.8)	28 (39.4)	43 (60.6)	11 (15.5)	60 (85.5)	27 (37.5)	45 (62.5)	29 (40.3)	43 (59.7)	13 (23.6)	42 (76.4)
IV			39 (72.2)	15 (27.8)	22 (40.7)	32 (59.3)	12 (22.2)	42 (77.8)	16 (29.6)	38 (70.4)	16 (29.6)	38 (79.4)	10 (23.8)	32 (76.2)
**Lymphatic invasion**	n	p	226	0.676	226	0.053	225	0.333	226	0.577	226	0.500	165	0.717
pL0			75 (64.1)	42 (35.9)	49 (41.9)	68 (58.1)	13 (11.2)	103 (88.8)	44 (37.0)	75 (63.0)	53 (44.5)	66 (55.5)	22 (25.9)	63 (74.1)
pL1			73 (66.9)	36 (33.1)	32 (29.4)	77 (70.6)	18 (16.5)	91 (83.5)	35 (32.7)	72 (67.3)	42 (39.3)	65 (60.7)	18 (22.5)	62 (77.5)
**Venous invasion**	n	p	225	0.827	225	0.666	224	0.373	225	**0.029**	225	0.213	164	0.164
pV0			130 (65.3)	69 (34.7)	73 (36.7)	126 (63.3)	26 (13.1)	172 (86.9)	74 (37.2)	125 (62.8)	81 (40.7)	118 (59.3)	37 (25.7)	107 (74.3)
pV1			18 (69.2)	8 (30.8)	8 (30.7)	18 (69.3)	5 (19.2)	21 (80.8)	4 (15.4)	22 (84.6)	14 (53.8)	12 (46.2)	2 (10.0)	18 (90.0)
**Tumor grade**	n	p	225	1.000	225	1.000	225	**0.027**	226	0.402	226	0.587	165	0.719
G1 / G2			64 (65.3)	34 (34.7)	37 (37.8)	61 (62.2)	7 (7.1)	91 (92.9)	39 (39.8)	59 (60.2)	43 (43.9)	55 (56.1)	19 (27.1)	51 (72.9)
G3 / G4			84 (66.1)	43 (33.9)	47 (36.7)	80 (63.3)	22 (17.3)	105 (82.7)	43 (33.6)	85 (66.4)	51 (39.8)	77 (60.2)	23 (24.2)	72 (75.8)
**Survival**	n	p	226	0.182	226	0.332	225	**0.030**	227	0.875	227	**0.005**	166	0.901
median ± SD [95%CI]			15.6 ± 2.1 [11.5-19.8]	22.4 ± 5.3 [12.0-32.7]	22.6 ± 3.1 [16.6-28.6]	14.7 ± 2.0 [10.7-18.6]	8.6 ± 5.9 [0.0-20.1]	17.9 ± 2.1 [13.7-22.1]	15.6± 2.1 [11.5-19.8]	17.9 ± 2.7 [12.7-23.2]	27.1 ± 7.4 [12.5-41.6]	13.4 ± 1.4 [10.7-16.1]	17.9 ± 3.2 [11.6-24.3]	15.4 ± 2.5 [10.5-20.3]

"n" detones numbers of patients. "p" denotes p-value. "SD" denotes standard deviation.

p-values printed in bold denote statistically significant correlations.
