# Supplementary material for: Integrins αvβ3 and αvβ5 as prognostic, diagnostic, and therapeutic targets in gastric cancer
Source: Gastric Cancer. 2014 Oct 15;18(4):784–95. doi: 10.1007/s10120-014-0435-2 (PMC4572058; doi:10.1007/s10120-014-0435-2)
Supplement: Supplementary file 2 — Supplementary material 2 (PDF 51 kb) [file 10120_2014_435_MOESM2_ESM.pdf]

Supplemental Table 2 Correlation between integrin expression in diffuse type gastric cancer and clinico-pathological patient characteristics

Diffuse Phenotype			αvβ3						αvβ5					
			Tumor Cells		Stroma Cells		Endothelium		Tumor Cells		Stroma Cells		Endothelium	
			Negative [n (%)]	Positive [n (%)]	Negative [n (%)]	Positive [n (%)]	Negative [n (%)]	Positive [n (%)]	Negative [n (%)]	Positive [n (%)]	Negative [n (%)]	Positive [n (%)]	Negative [n (%)]	Positive [n (%)]
**Gender**	n	p	144	1.000	144	1.000	144	0.854	140	1.000	141	0.360	33	0.686
Men			50 (82.0)	11 (18.0)	34 (55.7)	27 (44.3)	17 (27.9)	44 (72.1)	36 (61.0)	23 (39.0)	48 (80.0)	12 (20.0)	7 (30.4)	16 (69.6)
Women			68 (81.9)	15 (18.1)	46 (55.4)	37 (44.6)	25 (30.1)	58 (69.9)	49 (60.5)	32 (39.5)	70 (86.4)	11 (13.6)	2 (20.0)	8 (80.0)
**Localization**	n	p	144	0.750	144	1.000	144	0.589	140	0.458	141	0.198	33	1.000
Proximal stomach			15 (78.9)	4 (21.1)	11 (57.9)	8 (42.1)	4 (21.1)	15 (78.9)	10 (52.6)	9 (47.4)	14 (73.7)	5 (26.3)	3 (30.0)	57 (70.0)
Distal stomach			103 (82.4)	22 (17.6)	69 (55.2)	56 (44.8)	38 (30.4)	87 (69.6)	75 (62.0)	46 (38.0)	104 (85.2)	18 (14.8)	6 (26.1)	17 (73.9)
**pT-category**	n	p	144	0.432	144	0.501	144	0.474	140	0.539	141	0.265	33	0.524
pT1a			6 (85.7)	1 (14.3)	3 (42.9)	4 (57.1)	1 (14.3)	6 (85.7)	2 (66.7)	1 (33.3)	4 (100.0)	0 (0.0)	0 (0.00)	0 (0.00)
pT1b			4 (80.0)	1 (20.0)	1 (20.0)	4 (80.0)	1 (20.0)	4 (80.0)	2 (33.3)	4 (66.7)	5 (83.3)	1 (16.7)	0 (0.00)	0 (0.00)
pT2			12 (100)	0 (0.00)	6 (50.0)	6 (50.0)	3 (25.0)	9 (75.0)	7 (63.6)	4 (36.4)	9 (81.8)	2 (18.2)	2 (33.3)	4 (66.7)
pT3			45 (83.3)	9 (16.7)	30 (55.6)	24 (44.4)	13 (24.1)	41 (75.9)	30 (55.6)	24 (44.4)	44 (81.5)	10 (18.5)	5 (31.3)	11 (68.7)
pT4a			35 (74.5)	12 (25.5)	27 (57.4)	20 (42.6)	15 (31.9)	32 (68.1)	30 (63.8)	17 (36.2)	37 (78.7)	10 (21.3)	1 (11.1)	8 (88.9)
pT4b			16 (84.2)	3 (15.8)	13 (68.4)	6 (31.6)	9 (47.4)	10 (52.6)	14 (73.7)	5 (26.3)	19 (100)	0 (0.00)	1 (50.0)	8 (50.0)
**pN-category**	n	p	142	0.630	142	0.283	142	0.617	138	0.266	139	0.832	31	0.368
pN0			27 (87.1)	4 (22.9)	14 (45.2)	17 (54.8)	7 (22.6)	24 (77.4)	13 (43.3)	17 (56.7)	23 (76.7)	7 (23.3)	1 (10.0)	9 (90.0)
pN1			19 (86.4)	3 (13.6)	14 (63.6)	8 (36.4)	7 (31.8)	15 (68.2)	16 (76.2)	5 (23.8)	20 (90.9)	2 (9.1)	1 (16.7)	5 (83.3)
pN2			18 (72.0)	7 (28.0)	13 (52.0)	12 (48.0)	8 (32.0)	17 (68.0)	17 (70.8)	7 (29.2)	21 (87.5)	3 (12.5)	3 (42.9)	4 (57.1)
pN3 (a/b)			52 (81.3)	12 (18.7)	37 (57.8)	27 (42.2)	19 (29.7)	45 (70.3)	37 (58.7)	26 (41.3)	52 (82.5)	11 (17.5)	3 (37.5)	5 (62.5)
**Stage (UICC)**	n	p	144	0.654	144	0.420	144	0.473	140	0.803	141	0.492	30	0.569
IA			8 (80.0)	2 (20.0)	3 (30.0)	7 (70.0)	2 (20.0)	8 (80.0)	4 (44.4)	5 (56.6)	8 (88.9)	1 (11.1)	0 (0.00)	0 (0.00)
IB			6 (100)	0 (0.00)	3 (50.0)	3 (50.0)	0 (0.00)	6 (100)	2 (40.0)	3 (60.0)	4 (66.7)	2 (33.3)	1 (33.3)	2 (66.7)
IIA			13 (92.9)	1 (7.1)	8 (57.1)	6 (42.9)	5 (35.7)	9 (64.3)	7 (54.8)	6 (46.2)	11 (84.6)	2 (15.4)	0 (0.00)	5 (100)
IIB			11 (50.0)	11 (50.0)	8 (50.0)	8 (50.0)	2 (12.5)	14 (87.5)	10 (66.7)	5 (33.3)	11 (73.3)	4 (26.7)	2 (33.3)	4 (66.7)
IIIA			9 (75.0)	3 (25.0)	6 (50.0)	6 (50.0)	3 (25.0)	9 (75.0)	7 (58.3)	5 (41.7)	11 (91.7)	1 (8.3)	1 (33.3)	2 (66.7)
IIIB			23 (85.2)	4 (14.8)	15 (55.6)	12 (44.4)	9 (33.3)	18 (66.7)	16 (57.1)	12 (42.9)	21 (75.0)	7 (25.0)	3 (60.0)	2 (30.0)
IIIC			19 (70.4)	8 (29.4)	20 (74.1)	7 (25.9)	10 (37.0)	17 (63.0)	19 (70.4)	8 (29.6)	24 (88.9)	3 (11.1)	1 (33.3)	2 (66.7)
IV			26 (81.3)	6 (18.7)	17 (53.1)	15 (46.9)	11 (34.4)	21 (65.6)	20 (64.5)	11 (35.5)	28 (90.3)	3 (9.7)	1 (20.0)	4 (80.0)
**"Kiel"-Stage**	n	p	144	0.641	144	0.413	144	0.633	140	0.288	141	0.299	33	0.141
I			8 (80.0)	2 (20.0)	3 (30.0)	7 (70.0)	2 (20.0)	8 (80.0)	4 (44.4)	5 (56.6)	8 (88.9)	1 (11.1)	0 (0.00)	0 (0.00)
II			17 (89.5)	2 (10.5)	9 (47.4)	10 (52.6)	3 (15.8)	16 (84.2)	8 (42.1)	11(57.9)	13 (68.4)	6 (31.6)	1 (11.1)	8 (88.9)
IIIA			14 (93.3)	1 (6.7)	9 (60.0)	6 (40.0)	4 (26.7)	11 (73.3)	10 (71.4)	4 (28.6)	13 (86.7)	2 (13.3)	1 (14.3)	6 (85.7)
IIIB			35 (81.4)	8 (18.6)	24 (55.8)	19 (44.2)	14 (32.6)	29 (67.4)	26 (61.9)	16 (38.1)	34 (81.0)	8 (19.0)	6 (54.5)	5 (45.5)
IV			44 (77.2)	13 (22.8)	35 (61.4)	22 (38.6)	19 (33.3)	38 (66.7)	37 (66.1)	19 (33.9)	50 (89.3)	6 (10.7)	1 (14.3)	6 (85.7)
**Lymphatic invasion**	n	p	140	0.076	140	0.865	140	0.714	136	1.00	137	0.649	32	0.132
pL0			56 (88.9)	7 (11.1)	34 (53.9)	29 (46.1)	20 (31.7)	43 (68.3)	36 (60.0)	24 (40.0)	52 (85.2)	9 (14.8)	3 (16.7)	15 (83.3)
pL1			59 (76.6)	18 (23.4)	43 (55.8)	34 (44.2)	22 (28.6)	55 (71.4)	46 (60.5)	30 (39.5)	62 (81.6)	14 (18.4)	6 (42.9)	8 (57.1)
**Venous invasion**	n	p	140	0.152	140	1.000	140	1.000	136	0.741	137	0.175	32	0.073
pV0			110 (83.3)	22 (16.7)	73 (55.3)	59 (44.7)	40 (30.3)	92 (69.7)	77 (60.6)	50 (39.4)	108 (84.4)	20 (15.6)	7 (23.3)	23 (76.7)
pV1			5 (62.5)	3 (37.5)	4 (50.0)	4 (50.0)	2 (25.0)	6 (75.0)	5 (55.6)	4 (44.4)	6 (66.7)	3 (33.3)	2 (100)	0 (0.00)
**Tumor grade**	n	p	141	1.000	141	1.000	141	1.000	137	0.564	138	1.000	32	n. c.
G1 / G2			2 (100)	0 (0.00)	1 (50.0)	1 (50.0)	0 (0.00)	2 (100)	1 (33.3)	2 (66.7)	3 (100)	0 (0.00)	0 (0.00)	0 (0.00)
G3 / G4			113 (81.3)	26 (18.7)	77 (55.4)	62 (44.6)	40 (28.8)	99 (71.2)	81 (60.4)	53 (39.6)	112 (83.0)	23 (17.0)	9 (28.1)	23 (71.9)
**Survival**	n	p	138	0.731	138	0.519	138	0.435	134	0.539	135	0.229	126	0.928
median ± SD [95%CI]			12.9 ± 1.9 [9.2-16.7]	17.5 ± 10.5 [0.0-38.1]	12.5 ± 2.3 [8.4-17.3]	15.5 ± 6.1 [10.7-18.6]	11.8 ± 1.9 [7.9-15.5]	15.5 ± 2.6 [10.5-20.5]	12.8 ± 3.3 [6.3-19.4]	14.2 ± 2.1 [10.1-18.2]	14.2 ± 1.9 [10.4-17.9]	10.6 ± 7.8 [0.0-25.8]	17.1 ± 4.6 [8.0-26.1]	14.1 ± 2.2 [9.7-18.5]

"n" detones numbers of patients. "p" denotes p-value. "SD" denotes standard deviation.
