# Supplementary material for: Integrins αvβ3 and αvβ5 as prognostic, diagnostic, and therapeutic targets in gastric cancer
Source: Gastric Cancer. 2014 Oct 15;18(4):784–95. doi: 10.1007/s10120-014-0435-2 (PMC4572058; doi:10.1007/s10120-014-0435-2)

Online Resource 3 Evaluation and staining results of E-cadherin

Evaluation of E-cadherin Immunostaining

The quantity and intensity of immunoreactivity of E-cadherin in tumor cells was evaluated by applying an immunoreactivity scoring system (IRS) that consists of two components. Category A documented the intensity of immunostaining as 0 (no immunostaining), 1 (weak), 2 (moderate), and 3 (strong). Category B documented the percentage of immunoreactive cells as 0 (no immunoreactive cells), 1 (few scattered immunoreactive cells, <1%), 2 (1% to 10%), 3 (11% to 50%), 4 (51% to 80%), and 5 (>80%). The addition of category A and B resulted in an immunoreactivity scoring system ranging from 0 to 8 for each individual case.

Staining results of E-cadherin

E-cadherin was expressed in 404 of 443 cases (91.2%) in tumor cells. The percentage of stained tumor cells ranged from 0 to 5 (median 4), the staining intensity ranged from 0 to 3 (median 3). The tumor cell IRS ranged from 0 to 8 (median 6). Dichotomized by the median, 119 cases (26.9%) were classified as positive and 324 cases (73.1%) were classified as negative.

Score distribution for E-cadherin:

Cases	valid (%)	missing (%)	Distribution of score [n(%)]									Median Score	Split at median	
			0	1	2	3	4	5	6	7	8		negative [n(%)]	positive [n(%)]
482	443 (91.9)	39 (8.1)	36 (8.1)	0 (0)	4 (0.9)	11 (2.5)	13 (2.9)	54 (11.2)	85 (19.2)	121 (27.3)	119 (26.9)	7	324 (73.1)	119 (26.9)

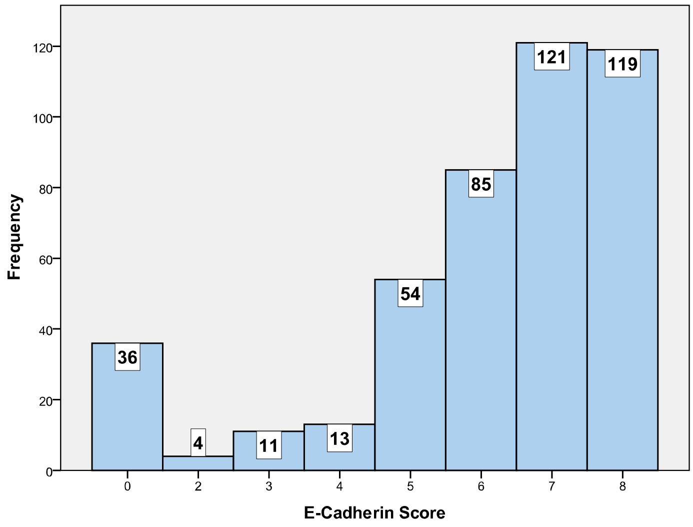

Supplement: Supplementary file 3 — Supplementary material 3 (PDF 51 kb) [file 10120_2014_435_MOESM3_ESM.pdf]
